# Common mechanisms and holistic care in atherosclerosis and osteoporosis

**DOI:** 10.1186/s13075-018-1805-7

**Published:** 2019-01-10

**Authors:** Zoltán Szekanecz, Hennie G. Raterman, Zsófia Pethő, Willem F. Lems

**Affiliations:** 1 Division of Rheumatology, Department of Internal Medicine, Faculty of Medicine, University of Debrecen, Nagyerdei street 98, Debrecen, 4032 Hungary; 2 Department of Rheumatology, Northwest Clinics, Wilhelminalaan 12, Alkmaar, JD 1815 The Netherlands; 3 Amsterdam Rheumatology and Immunology Centre, Location VU University Medical Centre, Amsterdam 1007 MB, Amsterdam, 1104 LP The Netherlands

**Keywords:** Atherosclerosis, Osteoporosis, Bone loss, Inflammation, Risk factors, DXA, Rheumatoid arthritis

## Abstract

Cardiovascular (CV) disease and osteoporosis (OP) have become increasing challenges in the aging population and even more in patients with inflammatory rheumatic diseases, such as rheumatoid arthritis, spondyloarthropathies, and systemic lupus erythematosus. In this review, we discuss how the epidemiology and pathogenesis of CV events and OP are overlapping. Smoking, diabetes mellitus, physical inactivity as conventional risk factors as well as systemic inflammation are among the modifiable risk factors for both CV events and bone loss. In rheumatic patients, systemic “high-grade” inflammation may be the primary driver of accelerated atherogenesis and bone resorption. In the general population, in which some individuals might have low-grade systemic inflammation, a holistic approach to drug treatment and lifestyle modifications may have beneficial effects on the bone as well as the vasculature. In rheumatic patients with accelerated inflammatory atherosclerosis and bone loss, the rapid and effective suppression of inflammation in a treat-to-target regime, aiming at clinical remission, is necessary to effectively control comorbidities.

## Introduction

Multimorbidity may become an increasing challenge in society [1]. In a study of more than 1.7 million individuals in Scotland, 42% had one or more morbidities and 23% were multimorbid [2].

In the last decades, both cardiovascular (CV) disease (CVD) and osteoporosis (OP) have been acknowledged as crucial health problems. Both diseases have a major impact on daily clinical functioning and quality of life and most importantly on life expectancy compared with the general population. Thus, CVD and OP should be considered major health issues [1–3].

CVD and OP may occur simultaneously in the general population under non-inflammatory conditions. Both CVD and OP have also been associated with autoimmune and inflammatory rheumatic diseases, such as rheumatoid arthritis (RA), spondyloarthropathies (SpAs), or systemic lupus erythematosus (SLE) [1, 4–15]. Immuno-inflammatory processes may accelerate atherosclerosis and bone loss under non-inflammatory conditions as well as in inflammatory rheumatic diseases [4, 16–18]. Immunosuppression including targeted therapies aiming at clinical remission may improve CVD and OP secondary to rheumatic diseases [4–6, 19].

**Fig. 1:**
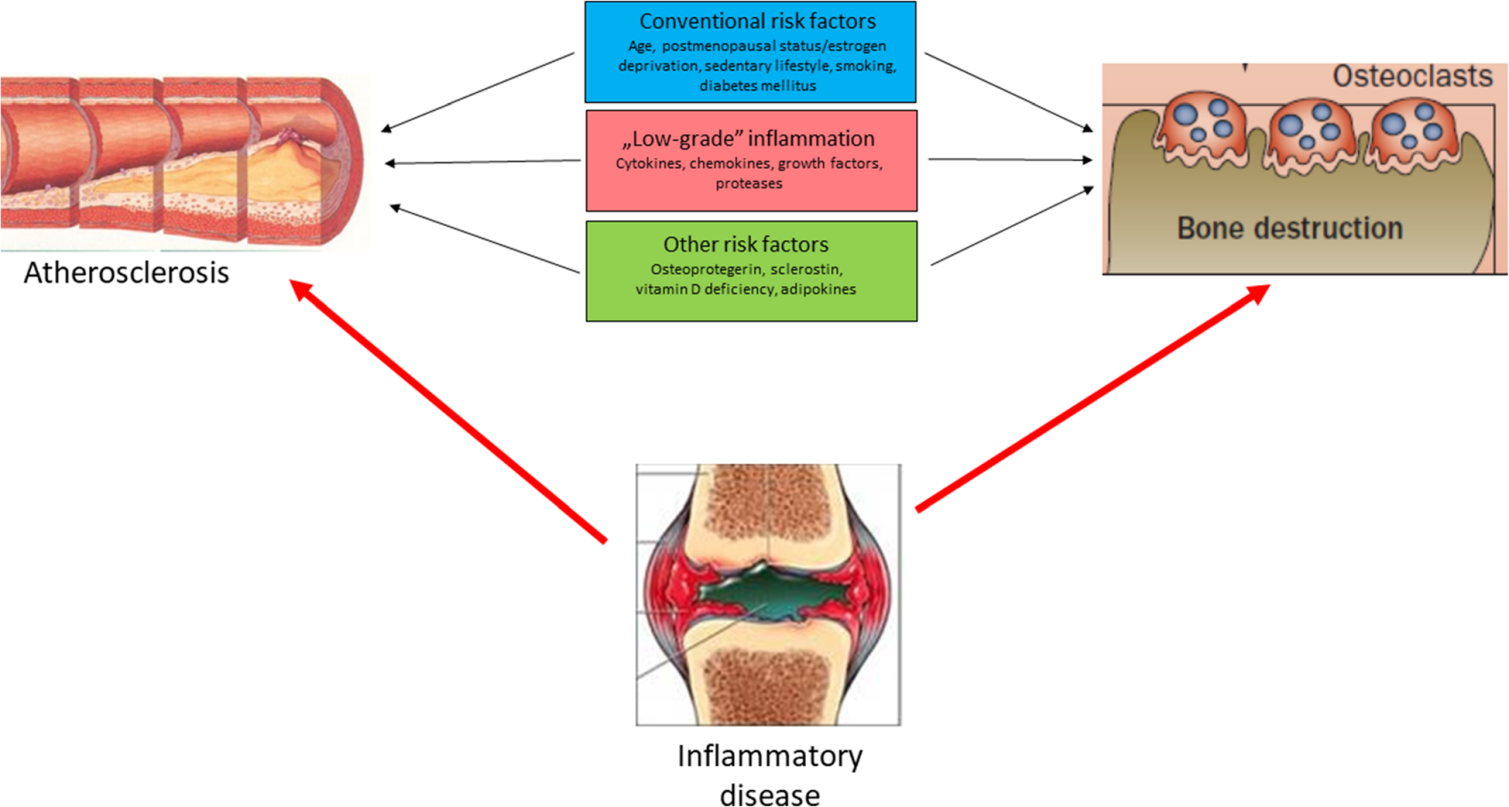
The “Bermuda triangle” of atherosclerosis, osteoporosis, and inflammation. Under non-inflammatory states, common conventional risk factors and “low-grade” inflammation may link atherogenesis and bone loss. In case of an underlying inflammatory disease, such as rheumatoid arthritis, “high-grade” systemic inflammation may perpetuate the development of atherosclerosis and osteoporosis

In this review, we will first briefly discuss the common pathogenic mechanisms in atherosclerosis and bone loss. As numerous cells and mediators are implicated in these processes, we will give only a general overview of these processes. Then we will discuss the two sides of the story: clinical and epidemiological evidence of increased CV risk in OP and bone loss and fragility fractures (FFs) in patients with CVD. As both CVD and OP are highly accelerated by systemic inflammation and autoimmunity, we will briefly present the “Bermuda triangle” of CVD, OP, and inflammatory rheumatic diseases (Fig. 1). We will choose RA as a prototype as the greatest amount of information is available on this disease. Finally, we will present some evidence that atherosclerosis, OP, and—if present—inflammation may be simultaneously and effectively targeted. The aim of this review is to describe the most relevant common mechanisms between bone loss and atherosclerosis under both non-inflammatory and inflammatory conditions. We also wish to put forward the idea of a holistic approach during disease prevention and therapy. We want to present a comprehensive review that discusses most aspects of this topic. Therefore, owing to space limitations, not all issues will be discussed in great detail.

### Common mechanisms in the development of atherosclerosis and bone loss

#### Conventional risk factors

Atherosclerosis/CVD and OP may share numerous risk factors (Table 1). Some conventional risk factors are relevant for both OP FF and CV events. Aging is by far the most important, but postmenopausal status or estrogen deprivation (or both), physical inactivity, and smoking also play major roles [20, 21].

**Table 1:** The most imprtant risk factors for atherosclerosis and osteoporosis^*^

	Atherosclerosis	Osteoporosis
Age	+++ [21, 24]	+++ [21, 24]
Dyslipidemia	+ [20]	± [20]
Hypertension	+ [20]	± [20]
Smoking	+ [21, 24]	+ [21, 24]
Diabetes mellitus	+ [22]	+ [22]
Sedentary lifestyle/immobility	+ [21, 24]	+ [21, 24]
Vitamin D deficiency	± [42]	+ [42]
Postmenopausal status/estrogen deprivation/premature menopause	+ [21, 24]	+ [21, 24]
“Low-grade inflammation”	++ [4, 5, 17, 18]	++ [6, 16]
Falls	–	+ [27]
Inadequate calcium intake	–	+ [21]
Alcohol consumption	–	+ [21]
Hypertension	+ [21]	–
Stress	+ [21]	–

^*^References in [ ]

In addition, diabetes mellitus (DM) not only is a well-known risk factor for CVD [22] but also has a negative effect on bone strength and increased incidence of FF, as has been shown recently [23]. There is evidence that type 1 DM (T1DM) is associated with lower bone mass, lack of insulin and insulin-like growth factor-1, and increased levels of pro-inflammatory cytokines leading to FF. In T2DM, in spite of increased bone mass, bone strength is decreased because of decreased bone formation, accumulation of advanced glycation end products (AGE), and cortical porosity. The paradox of higher bone mineral density (BMD) with increased FF risk in T2DM may also be explained by more frequent falls due to, among other things, diabetic retinopathy and polyneuropathy in addition to the poorer bone quality [23].

Additional conventional CV risk factors also associated with increased risk of low BMD may include dyslipidemia and hypertension [20]. However, the current evidence supporting the role of these factors in linking CVD and OP is not conclusive [20].

#### Inflammatory risk factors

In addition to traditional risk factors, systemic inflammation has been implicated in both atherosclerosis and bone loss. Inflammatory cells, acute phase reactants (for example, C-reactive protein, CRP; erythrocyte sedimentation rate, ESR), several pro-inflammatory cytokines, chemokines, and growth factors and other mediators—including osteoprotegerin (OPG), receptor activator of nuclear factor kappa B ligand (RANKL), and sclerostin (SOST)—are associated with both events [5, 16–18, 21, 24, 25].

“Low-grade inflammation”—including immune cells (T and B cells, macrophages, and endothelial cells) and inflammatory mediators—plays an important role in the pathogenesis of both atherosclerosis and OP. Furthermore, inflammatory rheumatic diseases, such as RA, SpA, or SLE, have been associated with secondary atherosclerosis, increased CV morbidity and mortality, and increased bone loss [4, 5, 7, 8, 18, 19, 21]. As described later, in such “high-grade inflammatory” states, both atherosclerosis and bone loss are even further accelerated, and treatment of the underlying inflammatory disease, primarily by targeted therapy, may have beneficial effects on the bone and vasculature [4, 6, 19, 26]. In general, among inflammatory mediators, CRP, pro-inflammatory cytokines (for example, tumor necrosis factor alpha [TNF-α] and interleukin 1 (IL-1), IL-6, IL-17, and IL-12/IL-23), some chemokines, growth factors, adipokines, and the renin–angiotensin–aldosterone system are important factors of atherogenesis and bone resorption [5, 6, 17, 18, 21, 24, 27–29]. For example, elevated CRP, TNF-α, and IL-6 levels have been associated with both myocardial infarction (MI) and non-traumatic FF [21, 29, 30].

Adipokines—primarily adiponectin, leptin, resistin, and chemerin—have been implicated in atherosclerosis under both inflammatory and non-inflammatory conditions [31, 32]. Various adipokines may also be involved in bone remodeling [31, 33]. For example, Varri et al. [34] performed a study on 290 postmenopausal women in Finland, in which adiponectin levels were inversely correlated with total body and femoral neck BMD but showed no relationship with coronary artery calcification (CAC) or carotid artery intima-media thickness (cIMT). In addition, leptin levels were associated with CAC [34].

The activated renin–angiotensin system (RAS) promotes atherogenesis. Angiotensin II also activates osteoclasts, while angiotensin-converting enzyme inhibitors may increase BMD and reduce FF risk [24, 35].

#### Factors primarily involved in bone metabolism

OPG and SOST are known factors related to bone biology. However, they have also been associated with CV events [36, 37]. OPG is a decoy receptor for RANKL that inhibits osteoclast activation and bone resorption [37]. In addition, OPG has been implicated in vascular calcification, matrix rearrangement, diabetic retinopathy, and most importantly in atherosclerosis, plaque destabilization, and CVD [36]. RANKL is overexpressed in vulnerable atherosclerotic plaques and may be able to reduce the risk of plaque rupture [24]. OPG has been related to CV morbidity and mortality [24, 38].

SOST is a known inhibitor of Wnt-mediated osteoblast activation and bone formation. Thus, SOST is involved in bone resorption and inflammatory bone loss [37, 39]. SOST has been associated with CV mortality in patients on hemodialysis [21]. Recently, SOST has been identified as a possible marker for early atherosclerosis [40].

Bone morphogenetic proteins (BMPs) induce mesenchymal cell differentiation toward the osteoblastic lineage. Various BMPs, primarily BMP-2 and BMP-7, stimulate collagen synthesis and are involved in bone formation. BMPs have been implicated in atherosclerosis. BMP-2 is expressed by vascular endothelial and smooth muscle cells. BMP expression is upregulated in the atherosclerotic plaques. Moreover, vascular BMP-2 expression is regulated by pro-inflammatory stimuli, such as TNF-α. BMP-4 has been associated with atherosclerosis and hypertension [24, 41].

Vitamin D may be considered both traditional and immuno-inflammatory risk factors for atherosclerosis with OP. Vitamin D deficiency has been implicated in bone loss and also in atherosclerosis [42, 43]. With regard to CVD, vitamin D deficiency may indirectly promote atherosclerosis on the basis of its relationship with DM, dyslipidemia, and hypertension. However, the exact mechanisms through which low vitamin D levels may directly lead from endothelial dysfunction to MI or stroke are not yet fully understood. Recent studies suggested that pathways potentially targeted by vitamin D include vascular muscle contractility, inflammatory mechanisms, oxidative stress, and the uptake of cholesterol by macrophages [44, 45].

Parathyroid hormone (PTH) is involved in age-related bone loss. PTH and PTH-related protein may also exert CV effects. Primary hyperparathyroidism has been associated with hypertension as well as increased CV morbidity and mortality [21, 24].

Fibroblast growth factor 23 (FGF-23) regulates phosphorous metabolism. Phosphorous may exert direct toxic effects on the vasculature. FGF-23 and one of its receptors, klotho, have been implicated in vascular calcification in patients on hemodialysis (see later). In addition, klotho deficiency and increased FGF-23 may lead to transformation of endothelial cells into osteoblast-like cells and mineralization. In contrast, high klotho expression inhibits vascular calcification [21, 46]. Even in individuals with normal renal function, high FGF-23 and low klotho levels may be associated with increased CV morbidity and mortality [21, 46] as well as OP and FF [21, 47].

Osteopontin (OPN) is an extracellular structural protein stimulated by 1,25-OH-vitamin D_3_. OPN promotes bone resorption via several molecular mechanisms. OPN is an inhibitor of ectopic calcification and vascular calcification [24, 48].

Cathepsin K exerts an important role in osteoclast activation and extracellular matrix degradation. Cathepsin K stimulates the degradation of type I and II collagens and osteonectin. It induces bone loss. Moreover, disruption of cathepsin K reduces plaque formation and the progression of atherosclerosis [24, 49].

#### Vascular calcification

Vascular medial wall calcification has been associated mainly with chronic kidney disease (CKD) [50]. The process involves impaired calcium and phosphate homeostasis, dysregulated PTH production, and abnormal calcium sensing [50, 51]. Recent studies have evaluated the role of Runx2, RANKL, OPG, and FGF-23 in CKD-associated vascular calcification [50, 51]. For example, as discussed above, high serum FGF-23, high phosphorous, and low klotho levels have been associated with increased arterial calcification in patients on hemodialysis [21]. Arterial calcification in CKD has been associated with low BMD [52] as well as impaired trabecular bone microarchitecture [53].

#### Surrogate markers indicating a relationship between atherosclerosis and bone loss

Certainly, the major issue is whether there is epidemiological and clinical evidence for increased CV risk in OP and increased risk of OP and FF in patients with CVD. Studies with significant clinical endpoints (MI, stroke, FF, and even mortality) are necessary to answer these two questions. It is important to note that in addition to clinical association studies described later, preliminary investigations using surrogate markers of subclinical CVD (for example, cIMT and CAC) were conducted in order to demonstrate associations with BMD. For example, Barengolts et al. [54] assessed coronary calcium burden by electron-beam computed tomography (CAC) and lumbar spine BMD in 45 postmenopausal women. Higher calcium scores were associated with lower BMD [54]. Campos-Obando et al. [55] assessed BMD and CAC during a mean of 6.4 years of follow-up in the Rotterdam Study. Increased BMD loss was associated with higher CAC scores in women but not in men. On the other hand, no associations between CAC and fracture risk could be observed [55]. Shaffer et al. [56] correlated cIMT and BMD in 535 women and 335 men. In the subset of individuals older than 60 years, BMD inversely correlated with cIMT [56]. Finally, Delgado-Frias et al. [57] found an inverse correlation between OPG levels and brachial artery flow-mediated vasodilation (FMD), a marker of endothelial function in patients with RA. These studies using surrogate markers already suggest that atherosclerosis/CVD and bone loss/BMD may be associated.

In conclusion, numerous non-inflammatory (traditional) and inflammatory factors may link atherosclerosis and osteoporosis. Some of these factors may be used as surrogate markers of the two interrelated processes.

### Increased cardiovascular risk in osteoporosis

As mentioned in the previous section, OP and CVD have common pathophysiological links and risk factors [21, 24]. Epidemiological evidence has been found between an increased risk and prevalence of CVD in patients with OP [24]. In the recent meta-analysis (of 25 studies with 10,300 patients) performed by Ye et al. [58], the adjusted incidence of any atherosclerotic vascular abnormality was significantly increased in individuals with low BMD versus normal BMD (odds ratio (OR) 2.96) as well as in those with OP versus without OP (OR 2.45) [58]. Veronese et al. [59] performed a meta-analysis of 11 studies indicating that people with low BMD are at increased risk of developing CVD during follow-up with an adjusted hazard ratio (HR) of 1.33 [59]. Moreover, this meta-analysis observed that each decrease of BMD by one standard deviation (SD) increased the CVD risk by 16% (HR 1.16). In regard to specific CVD types, each BMD decrease by one SD corresponded to a consecutively increased risk of CV and cerebrovascular disease by 44% and 28%, respectively [59]. In contrast, Jin et al. [60] published data from the recent Korean STROBE (Strengthening the Reporting of Observational studies in Epidemiology) study. They could not completely confirm the observed association between cerebrovascular disease and OP, as vertebral or hip BMD was not associated with cerebral arterial disease [60].

With respect to associations between FF and CVD, Veronese et al. [59] reported a significant association between FF at baseline and subsequent development of CVD (HR 1.20). FF at baseline was also associated with an increased risk of cerebrovascular conditions and death due to CVD [59]. In contrast, in a much earlier study, Varosy et al. [61] unexpectedly found a decreased risk of subsequent coronary events in 2700 postmenopausal women with known CVD and skeletal fractures. In this study, the risk of developing further coronary events during 4 years’ follow-up was reduced by 25% in women with FF compared with those without fractures (HR 0.74). It is important to note that these patients already had prevalent CVD in addition to FF at baseline [61]. Therefore, the exact association between FF and later CVD needs to be determined by conducting larger prospective studies [62].

Some studies also assessed CV mortality in patients with OP. In the meta-analysis by Qu et al. [63], an increased risk of CV mortality was described in patients with OP. A significant relationship between low BMD at all sites and CV mortality was found. On the other hand, this study could not demonstrate an association between cerebrovascular mortality and OP [63]. In a prospective study of more than 700 individuals, Domiciano et al. [64] observed increased CVD mortality in patients with OP as defined by total hip T-score (HR 3.17). Moreover, this study suggested that a one-SD decrease of total hip BMD was a predictor for all-cause mortality in elderly people [64]. These findings are in line with some early studies that reported increased CV mortality in patients with OP [21, 24]. However, the recent meta-analysis by Veronese et al. [59] suggests that publication bias may have influenced some of the abovementioned results, so the precise HR of OP on CVD mortality remains to be elucidated [59].

In conclusion, even in non-inflammatory states and despite some controversial reports, there is increased risk of atherosclerosis and CVD in individuals with OP. Several pathogenic processes may link atherosclerosis to OP. Moreover, patients with OP should be routinely screened for atherosclerosis and CVD.

### Increased risk of osteoporosis and fragility fractures in cardiovascular disease

There is evidence suggesting that patients with CVD have an increased risk of bone loss and FF. Den Uyl et al. [65] performed a systematic review of seven population cohort studies on patients with subclinical CVD. Six of the highest-ranked studies that included a mean of 2000 patients indicated that subjects with prevalent subclinical CVD disease had higher risk for increased bone loss and FF compared with individuals without CVD. This was observed in both women and men. Both vertebral and hip FFs were observed. The overall HR/OR was 2.3 to 3.0. The data also suggested that subjects with low BMD had higher CV mortality rates and incident CV events than those with normal BMD. In this analysis, the most important risk factors were age, estrogen deficiency, and inflammation [65].

Sennerby et al. [66] followed almost 32,000 Swedish twins from the age of 50 years for a mean follow-up time of 20 years. None of the subjects had CVD at this age. The main outcome measure was time to hip fracture after diagnosis of CVD. The crude absolute rates of FF per 1000 person-years after the diagnosis of heart failure, stroke, peripheral arterial disease, and ischemic heart disease compared with no-CVD were 12.6, 12.6, 6.6, and 5.2 versus 1.2, respectively. The adjusted HRs of hip FF in these CVDs were 4.4, 5.1, 3.2, and 2.3, respectively. Thus, CVD was significantly associated with subsequent FF [66].

Gerber et al. [67] followed 3321 patients with incident MI and 3321 controls until an FF or death occurred. The overall FF HR in MI patients was 1.32. This HR increased according to time period: in 1979–1989, 1990–1999, and 2000–2006, the HRs were 0.81, 1.47, and 1.73, respectively [67]. These changes could be explained by increasing age over time as well as other environmental factors.

Finally, Pouwels et al. [68] assessed the risk of hip/femur FF after stroke. The adjusted ORs of FF in any stroke and hemorrhagic, ischemic or undefined stroke were 1.96, 1.94, 1.85, and 2.10, respectively, compared with those subjects who never experienced stroke [68].

In conclusion, there is increased risk of bone loss, OP, and FF in patients with CVD. A number of mechanisms may lead to bone loss in CVD. Furthermore, patients with CVD should be regularly screened for OP.

### Cardiovascular disease and osteoporosis in inflammatory rheumatic diseases

Patients with inflammatory rheumatic diseases not only may suffer from their disease but also may have comorbidities [1, 13]. The greatest amount of information has become available in RA [1, 4, 13]; therefore, we will mostly discuss this disease with implications for other inflammatory rheumatic diseases. For example, in the large COMORA (Comorbidities in Rheumatoid Arthritis) study of 4586 patients with RA in 17 countries, a high prevalence of comorbidities and their risk factors was found, but the variability between countries in the prevalence and also in the detection of comorbidities was highly variable [13]. In a recent prospective Swedish cohort of 950 patients with early RA, factors associated with the development of one or more comorbidities, including CVD, stroke, and OP, were analyzed. Disease activity and ESR were among the most common risk factors for comorbidities, indicating the importance of inflammation [25].

As already mentioned above, both CVD and OP have been associated with RA as well as other types of arthritis (for example, SpA) and autoimmune connective tissue diseases (for example, SLE) [1, 4–15, 69]. The risks of both CVD and FF are increased, roughly doubled in RA, carrying a large impact on the quality of life in these patients [4, 8, 13, 15, 69]. Again, the crucial question is whether these comorbidities, as described above, are also interrelated under inflammatory conditions. Relatively few studies have been carried out in RA or SLE assessing whether a fracture would be a risk factor for a CV event or CVD would increase OP or FF risk.

With respect to CVD and OP in arthritis and autoimmune patients, a recent population-based cohort study by Ni Mhuircheartaigh et al. [70] indicated a substantial increase of CVD development in RA patients with FF. In this study, 1171 incident RA patients compared with 1171 non-RA subjects with a sustained FF were followed for 12 years for further CVD development. In controls, FF was not associated with increased CV risk (HR 1.10–1.12). However, in RA subjects with FF, significantly increased CV risk was shown (HR 1.81 for FF and 1.80 for major OP fracture subjects) [70]. To the best of our knowledge, no other studies on FF and CVD development in rheumatic conditions have been performed.

With respect to non-FF studies, Popescu et al. [71] reported that patients with RA and CV comorbidity had lower total bone mass. Provan et al. [72] presented 15-year follow-up data from the Oslo RA Register. RA patients, who deceased from primary atherosclerotic death including both CV or cerebrovascular disease, were more often suffering from OP (57% versus 36%) and previous fractures (38% versus 21%) compared with those who did not die of CVD [72].

In other rheumatic diseases, such as SLE, the information on CV risk in OP is limited. The only studies published used surrogate markers for CVD, such as carotid ultrasound rather than clinical CVD data. Ramsey-Goldman and Manzi [14] reported associations between more carotid plaques or coronary calcification and low BMD in young patients with SLE.

Autoantibodies play a major role in the pathogenesis of RA and other autoimmune diseases. Anti-citrullinated protein antibodies (ACPAs) and rheumatoid factor (RF), which play an essential role in RA, may also be associated with atherogenesis and bone loss. Indeed, both ACPA and RF positivity have been associated with increased CV risk in RA [73]. Furthermore, ACPA and RF, even independently of inflammation, may synergistically induce bone resorption in RA [74].

In conclusion, inflammatory processes and underlying inflammatory diseases may enhance the development of both atherosclerosis and OP. “Accelerated” or “inflammatory” atherosclerosis and bone loss have been associated with RA and other inflammatory conditions. Therefore, such patients should be routinely screened for CVD and OP.

### Possibilities of parallel targeting of cardiovascular disease and osteoporosis

Although some anti-OP drugs may have favorable CV effects and inversely some vasoactive agents may have positive effects on the bone, it is more likely that in the case of arthritides and autoimmune diseases, treatment of the underlying disease may also improve CV and OP comorbidities [4, 6, 19]. There have been multiple studies in this field, so here we only briefly summarize general considerations.

Vitamin D has been considered an essential background treatment and preventative compound in OP. Vitamin D deficiency has also been associated with T1DM and CVD [42, 45]. Furthermore, vitamin D exerts numerous immunomodulatory properties and thus may be used in immuno-inflammatory diseases [42, 45].

Calcium supplementation has been implicated in the development of CVD in the general population, but this issue is still under debate. This association was suggested by some large studies (for example, NIH-AARP) but conflicted by others (for example, Framingham and the Women’s Health Initiative) [72, 75]. Interestingly, according to a recent study, calcium supplementation together with high (but not with low) ESR was associated with increased all-cause and CV mortality of patients with RA [72]. However, it is difficult to evaluate calcium supplementation as a CV risk in RA. It is possible that calcium was mostly prescribed to RA patients with OP who already have a high background risk for CVD.

Among anti-OP agents, bisphosphonates in some studies inhibited atherogenesis and significantly decreased serum low-density lipoprotein (LDL) and increased high-density lipoprotein (HDL) in postmenopausal women [20]. Yet the effect of bisphosphonates on CV risk is not clear, but a reduction in mortality was prescribed for zoledronic acid after a hip fracture [76]. As described above, OPG has been linked to vascular calcification and CVD [36]. Therefore, it is an important question whether RANKL inhibition by denosumab would affect the CV system. Samelson et al. [77] compared more than 2300 denosumab- or placebo-treated patients from the FREEDOM (Fracture Reduction Evaluation of Denosumab in Osteoporosis every 6 Months) trial. RANKL inhibition had no effect at all on the progression of arterial calcification or on the incidence of CV events [77].

On the other hand, statins and nitrates, agents used in vasculoprotection, may also have beneficial effects on the bone [20, 78]. Weaker evidence suggests that thiazide diuretics and β-blockers may also exert favorable effects on bone [78]. By contrast, some drugs, such as loop-acting diuretics and warfarin, may aggravate bone loss [78]. There have been very few prospective trials in this respect, so the effects of heart drugs on bone should be confirmed by future studies [78].

With respect to anti-rheumatic and anti-inflammatory drugs used to treat RA and other inflammatory rheumatic diseases, there has been a lot of controversy on the benefits versus risks of low-dose corticosteroid treatment. In general, corticosteroids indeed may be pro-atherogenic [79] and may stimulate bone loss [80]. However, in RA, the anti-inflammatory effects of low-dose corticosteroid treatment may be beneficial for vasculature and bone. The net effect of corticosteroids on CVD and OP may vary in different diseases and patients [4, 5, 26, 79–82]. According to a recent OP consensus paper by the ESCEO (European Society for Clinical and Economic Aspects of Osteoporosis and Osteoarthritis) [81] and the recent EULAR CV recommendations [4], short durations and moderate doses of glucocorticoids are recommended. In this case, corticosteroids are generally well tolerated and have a positive benefit/risk ratio. Yet patients on corticosteroid therapy should be regularly assessed for CV and FF risk and, if needed, should be treated [4, 80, 81].

Among conventional synthetic disease-modifying anti-rheumatic drugs (csDMARDs), methotrexate (MTX) may exert beneficial effects on bone and on the CV system primarily by controlling systemic inflammation [4, 5, 26, 82]. MTX has not only indirect but also direct effects on bone resorption [83] and lipid metabolism [5].

In brief, biologic DMARDs (bDMARD), primarily TNF inhibitors, may also decrease inflammatory atherogenesis [5, 17, 19, 84], may lower the risk of CVD [4, 5, 19, 82, 85, 86], and may halt periarticular and generalized bone loss [6, 26, 87, 88] in inflammatory rheumatic diseases. Moreover, according to a recent study, RA patients receiving TNF inhibitors had even lower risk of MI compared with those treated with csDMARDs [86]. In a recent prospective early RA cohort, the use of biologics was inversely associated with the risk of comorbidity development [25].

Finally, in addition to the EULAR recommendations on CVD management in rheumatic diseases [4] and the recent EULAR/European Federation of National Associations of Orthopaedics and Traumatology (EULAR/EFORT) recommendations on the management of FF [9], a EULAR initiative published “points to consider” for reporting, screening, and preventing comorbidities in patients with chronic inflammatory rheumatic diseases. This paper focuses on six comorbidities, including CVD and OP. These recommendations also include advice on lifestyle modifications in addition to drug therapy [89].

In conclusion, one can set up a holistic approach to the management of CVD and OP, especially in patients with inflammatory rheumatic diseases. The understanding of common pathogenic factors, as molecular targets, may help us to design novel strategies that combat both CV disease and bone loss.

## Conclusions

In an aging population, comorbidities such as CVD or OP occur more frequently. These comorbidities develop even more often in patients with inflammatory rheumatic diseases such as RA and SLE. These interactions are plotted in Fig. 1 as a “Bermuda triangle”. Although rheumatologists nowadays are successful in treating their patients according to the treat-to-target design (particularly in RA), it is obvious that this single disease framework may not cover all comorbidities; therefore, comorbidities are still suboptimally prevented, screened, and managed. In this review, we discussed how the epidemiology and pathogenesis of CV events and OP are strikingly overlapping. Theoretically, smoking, DM, and sedentary lifestyle as conventional risk factors and systemic inflammation are among the modifiable risk factors for both atherosclerosis and bone loss. A holistic approach to treatment may involve the use of drugs and lifestyle modifications that may have beneficial effects on bone as well as on vasculature. In RA patients with accelerated inflammatory atherosclerosis and bone loss, the rapid and effective suppression of inflammation by corticosteroids, csDMARDs, and bDMARDs in a treat-to-target manner aiming at clinical remission is necessary to effectively control comorbidities.

One of the limitations of the reviewed dataset is that most of the studies have been conducted in Caucasian women; thus, extrapolation to men and women of different genetic background is not possible. Another issue is that the associations of CVD and OP in patients with RA are highly dependent on the underlying systemic inflammation. Thus, it is not clear whether a similar relationship would be valid in modern times, when RA therapy is aiming at clinical remission. Finally, possibly the most important limitation is that almost no data have been presented on the relationship between cumulative disease activity, functional capacity and/or radiological damage and the risk of CV events and/or OP in patients with arthritis. For example, in the recent study by Ni Mhuircheartaigh et al. [70] discussed above, the risk of a CV event is 80% higher in RA patients after a fracture but is likely to be even higher in RA patients with high disease activity and lower in patients in clinical remission. Thus, there is still a lot of work to do both in research and in daily practice: the conduction of more prospective studies that assess the epidemiological, clinical, and pathophysiological characteristics of CVD and OP in parallel is urgently needed, but optimal treatment aiming at remission and advocating lifestyle factors in all our patients is also a challenge.

## Abbreviations

ACPA: Anti-citrullinated protein antibody
bDMARD: Biologic disease-modifying anti-rheumatic drug
BMD: Bone mineral density
BMP: Bone morphogenetic protein
CAC: Coronary artery calcium
cIMT: Carotid intima-media thickness
CKD: Chronic kidney disease
CRP: C-reactive protein
csDMARD: Conventional synthetic disease-modifying anti-rheumatic drug
CV: Cardiovascular
CVD: Cardiovascular disease
DM: Diabetes mellitus
DMARD: Disease-modifying anti-rheumatic drug
ESR: Erythrocyte sedimentation rate
EULAR: European League Against Rheumatism
FF: Fragility fracture
FGF-23: Fibroblast growth factor 23
HR: Hazard ratio
IL: Interleukin
MI: Myocardial infarction
MTX: Methotrexate
OP: Osteoporosis
OPG: Osteoprotegerin
OPN: Osteopontin
OR: Odds ratio
PTH: Parathyroid hormone
RA: Rheumatoid arthritis
RANKL: Receptor activator of nuclear factor kappa B ligand
RF: Rheumatoid factor
SD: Standard deviation
SLE: Systemic lupus erythematosus
SOST: Sclerostin
SpA: Spondyloarthritis
T1DM: Type 1 diabetes mellitus
TNF: Tumor necrosis factor
